# Antitumor Activity of Metformin in Combination with Binimetinib Against Melanoma Cells

**DOI:** 10.3390/ijms262311403

**Published:** 2025-11-25

**Authors:** Radosław Wolan, Joanna Wawszczyk, Arkadiusz Orchel, Małgorzata Kapral

**Affiliations:** 1 Department of Biochemistry, Faculty of Pharmaceutical Sciences in Sosnowiec, Medical University of Silesia in Katowice, Jedności 8, 41-200 Sosnowiec, Poland; radoslaw.wolan@sum.edu.pl (R.W.); jwawszczyk@sum.edu.pl (J.W.); 2 Department of Pharmacology, Faculty of Pharmaceutical Sciences in Sosnowiec, Medical University of Silesia in Katowice, Jagiellońska 4, 41-200 Sosnowiec, Poland; 3 Department of Biopharmacy, Faculty of Pharmaceutical Sciences in Sosnowiec, Medical University of Silesia in Katowice, Jedności 8, 41-208 Sosnowiec, Poland; aorchel@sum.edu.pl

**Keywords:** melanoma, metformin, binimetinib, melanoma treatment, drug combination

## Abstract

Malignant melanoma is skin cancer with high metastatic potential and resistance to treatment. Significant progress has been made following the introduction of its treatment kinase inhibitors such as binimetinib. Some studies suggested that the combination of metformin with BRAF/MEK inhibitors suppresses cancer cell growth and progression. This study aimed to assess the impact of metformin and binimetinib, both individually and in combination, on the growth, proliferation, and apoptosis of melanoma cells in vitro. The study also sought to predict potential interactions between metformin and binimetinib when co-administered employing in silico analysis. Cell growth and proliferation of melanoma cells exposed to metformin and binimetinib, alone and in combination, were determined by SRB and BRDU assays. Further investigations were performed using real-time RT-qPCR and ELISA method. These results revealed that the simultaneous use of metformin and binimetinib exerted an additive or synergistic inhibitory effect on melanoma cell growth. The concomitant effect of both compounds depended on the concentrations used and was caused by the reduced proliferation and/or increased apoptosis of melanoma cancer cells. In conclusion, the combination of metformin and binimetinib may have potential anticancer effects on melanoma cells; however, more studies are needed to elucidate the exact mechanisms of their combined action.

## 1. Introduction

Malignant melanoma represents a growing concern among skin cancers due to its rising incidence, currently comprising 1.7% of all newly diagnosed malignant neoplasms worldwide and 3.7% in Europe. Notably, the incidence of newly diagnosed melanoma cases has been consistently rising over an extended period, and predictive models indicate that this upward trend is likely to persist in the foreseeable future [1,2,3,4]. Malignant melanoma is a malignant tumor originating from melanocytes, pigment-producing cells that synthesize melanin [5]. A subset of melanomas (2–8%) are amelanotic melanomas, characterized by a complete or partial lack of melanin production [6]. While most melanomas arise de novo, malignant transformation can also occur in pre-existing pigmented lesions. Melanoma is considered the most dangerous form of skin cancer due to its high metastatic potential during disease progression [5]. Malignant melanoma additionally demonstrates resistance to treatment with cytostatic drugs. More effective therapeutic options include molecularly targeted therapies and immunotherapy, which have been introduced in recent years. The selection of an appropriate pharmacological treatment method depends on the presence of mutations in the *BRAF* gene, which occur in approximately 50% of cases. This mutation results in the hyperactivation of the intracellular MAP kinase signaling pathway, leading to excessive and uncontrolled cell proliferation [7,8]. For systemic treatment, patients with the *BRAF V600* mutation are treated with combination therapy using BRAF and MEK inhibitors, along with anti-PD-1 immunotherapy, typically in combination with ipilimumab, regardless of *BRAF* mutation status [9]. These therapies demonstrate effectiveness only in a subset of patients with metastatic melanoma, and in a limited number of cases, their use leads to disease remission. Consequently, there remains an ongoing need to explore new treatment options [10].

A promising avenue in the development of innovative therapies involves investigating new applications for existing drugs originally developed for other medical indications. One such compound is metformin, an antidiabetic drug that has been used since the 1960s. Long-term observations suggest that it may have a preventive role in cancer development, as well as exhibit direct anticancer activity [11,12,13,14,15,16,17]. The potential anticancer activity of metformin arises, on the one hand, from inhibition of Complex I of the mitochondrial respiratory chain which leads to activation of AMP-activated protein kinase (AMPK) [18,19,20]. On the other hand, metformin also inhibits insulin and insulin-like growth factor 1 (IGF-1) secretion, which in turn activates the AKT kinase. The activity of both kinases impedes intracellular signaling pathways through mTOR kinases, resulting in modulation of protein synthesis, suppression of cell proliferation, and induction of apoptosis [21,22,23].

Binimetinib is an anticancer drug used in combination therapy for malignant melanoma with a V600 mutation in the *BRAF* gene. It belongs to a class of small molecule reversible inhibitors targeting MEK1 and MEK2 kinases, which are integral components of the Ras/Raf/MEK/ERK signaling cascade [24]. This pathway is involved in transmitting signals from membrane receptors to enzymatic proteins that regulate fundamental cellular processes [25]. Furthermore, this signaling cascade is also linked with the PI3K pathway, which is influenced by metformin, and may function both as an effector and as an alternative signal transduction route [26]. The intracellular PI3K/AKT/mTOR and MAPK/ERK pathways cooperate in tumor growth and are known aberrantly activated in melanoma cells, contributing to enhanced cell proliferation and inhibition of apoptosis [27,28]. The convergence of intracellular signaling pathways targeted by metformin and binimetinib supports the rationale for exploring their synergistic antitumor potential. It has been shown that metformin can enhance the antitumor activity of another class of kinase inhibitors, specifically EGFR kinase inhibitors, in the treatment of lung cancer [29].

This study aimed to assess the impact of metformin and binimetinib, both individually and in combination, on the growth, proliferation, and apoptosis of amelanotic and melanotic melanoma cells in vitro. The study sought to predict potential interactions between metformin and binimetinib when co-administered, along with the identification of the pathways, diseases, and genes affected by the combined action of both drugs, employing in silico analysis.

## 2. Results

### 2.1. In Silico Analysis

A computational analysis was conducted to predict the potential effects of simultaneous treatment of cancer cells with metformin (Met) and binimetinib (Bin), utilizing ChemDIS-Mixture version 2.4. The ChemDIS-Mixture pathway tool enabled the prediction of possible pathways affected by treatment with both compounds alone and simultaneously. The results identified 327 pathways affected by metformin and 95 pathways influenced by binimetinib. Among these, 36 signaling pathways were shared by both. The adjusted *p*-values were calculated for metformin, binimetinib, and their combined application (Adj.P _Joint_). A lower Adj.P _Joint_ value suggests a potentially greater likelihood of interaction between the two compounds when used in combination therapy. Notably, many of these shared pathways are known to be active in cancer (e.g., MAPK, ErbB and VEGF signaling pathway). The selected pathways with the highest probability of interaction with both drugs are shown in Table 1.

Further analysis enabled the identification chemical-associated gene ontology (GO), which constitute 1204 biological processes are potentially affected by metformin, 197 genes influenced by binimetinib, and 61 overlapping genes that may serve as common targets for both agents. The most important of which are presented in Table 2. Genes selected as targets of both compounds encode proteins involved in essential cellular processes, including DNA replication (RNA polymerase II) and protein phosphorylation (MAPK cascade). The simultaneous use of metformin and binimetinib potentially affects fundamental cellular metabolic processes such as genes transcription and cells proliferation. The aforementioned genes undergo excessive and uncontrolled activation in numerous cancer cells, constituting a significant factor contributing to tumor development.

The ChemDIS Mixture Disease Ontology (DO) tool was employed to identify potential therapeutic indications for the combined use of metformin and binimetinib. The highest potential for interaction between tested compounds was observed in endocrine disorders and genetic diseases. Nevertheless, the analysis also indicated a strong likelihood of synergistic interactions between the two compounds in the context of various malignancies, notably gastrointestinal system cancer. Table 3 summarizes 20 of the DO cancer terms along with the adjusted *p*-values (Adj.P) associated with metformin, binimetinib and their combined application (Adj.P _Joint_). Furthermore, the findings suggest that the co-administration of metformin and binimetinib may also impact other cancer types, including hematological, prostate and lung cancers.

### 2.2. Impact of Metformin and Binimetinib on Cell Growth

**Figure 1:**
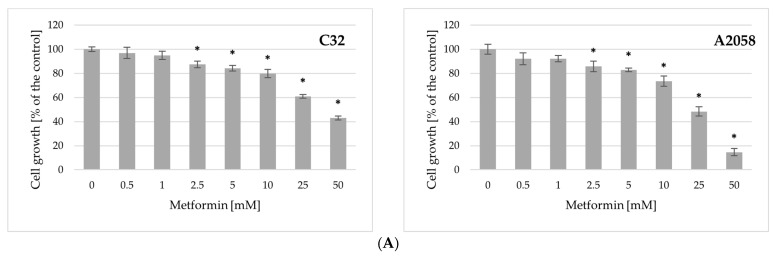

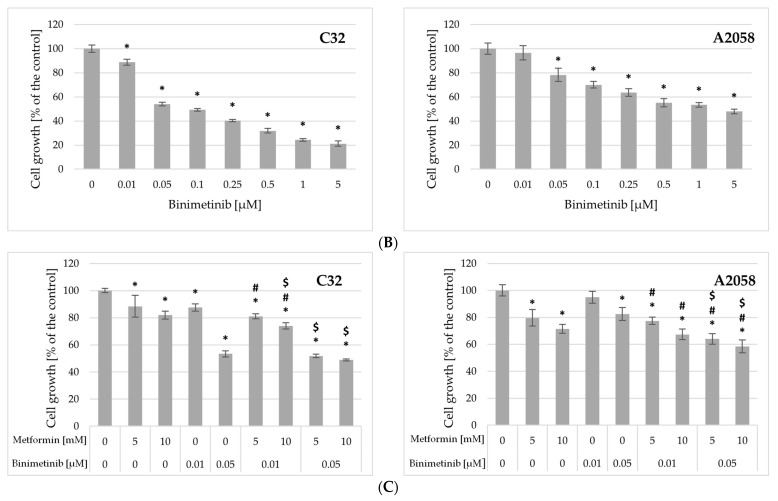
Influence of metformin (**A**), binimetinib (**B**) and metformin and binimetinib simultaneously (**C**) on the growth of C32 and A2058 cells after 72 h of treatment. Data are expressed as the means ± SD and are represented as percentages of the untreated control (100% growth) of three independent experiments involving six analyses for each sample. The statistical significance of differences was assessed by one-way ANOVA followed by the post hoc Tukey’s test; * *p* < 0.05 vs. control; $ *p* < 0.05 vs. metformin alone; # *p* < 0.05 vs. binimetinib alone.

The experiment focused first on investigating the effects of metformin and binimetinib used alone on the growth of C32 and A2058 melanoma cells after 72 h of treatment. The results obtained have shown that metformin at concentrations ≥ 2.5 mM significantly reduced cell growth of tested cell lines in a dose-dependent pattern (Figure 1A) The half-maximal inhibitory concentration (IC50) value was found to be 36.1 mM for C32 cells and 18 mM for A2058 cell, and reflected different levels of sensitivity of both cell lines to metformin. As shown in Figure 1B, binimetinib also markedly decreased the growth of the studied melanoma cells. The growth of C32 cells was significantly decreased at all drug concentrations used. Binimetinib at 0.01 mM concentration did not affect A2058 cell growth. However, it significantly decreased A2058 cell growth at concentrations of ≥0.05 mM. The IC50 values for binimetinib were 0.16 μM for the C32 cell line and 1.52 μM for the A2058 cell line. These results indicate that metformin exerted a stronger inhibitory effect on the growth of A2058 melanoma cells, while binimetinib exhibited greater efficacy against C32 melanoma cells.

The results of the analysis of both drugs on cell growth enabled the selection of appropriate concentrations of metformin and binimetinib for studying their combined effect on melanoma cells. To study the possible synergistic impact of metformin and binimetinib on the growth of C32 and A2058 melanoma cells, cell cultures were incubated with metformin (Met) and binimetinib (Bin) in different combinations (Met5 + Bin0.01; Met10 + Bin0.01; Met5 + Bin0.05 and Met10 + Bin0.05) and individually for 72 h. The growth of C32 and A2058 cells in cultures treated with both drugs simultaneously was significantly decreased in comparison to control (Figure 1C). Treatment of C32 cells with the combination of metformin and binimetinib at a concentration of 0.01 μM (Met5 + Bin0.01 and Met10 + Bin0.01) caused a statistically significant, stronger inhibitory effect on cell growth compared to the effect caused by binimetinib alone. In cultures treated with both drugs simultaneously (Met10 + Bin0.01, Met5 + Bin0.05 and Met10 + Bin0.05), a stronger inhibition of cell growth was observed compared to the effect of metformin alone.

The types of metformin and binimetinib interactions were determined using a Combination Index (CI). The findings revealed that the combination of metformin with higher concentrations of binimetinib (Met5 + Bin0.05 and Met10 + Bin0.05) had a synergistic effect on C32 cells growth and CI values were 0.52 and 0.56, respectively. The growth of C32 cells in cultures treated with both drugs decreased by 48.2% (Met5 + Bin0.05) and 51.1% (Met10 + Bin0.05) compared to untreated control cells. These cells are also characterized by the highest sensitivity to binimetinib and the combination of metformin and binimetinib. For A2058 cell line, synergism (Met5 + Bin0.05, CI = 0.68) or an additive effect (Met10 + Bin0.05, CI = 0.95) was observed. The growth of A2058 melanoma cells was reduced, respectively, by 36% and 41.5% in relation to control.

**Figure 2:**
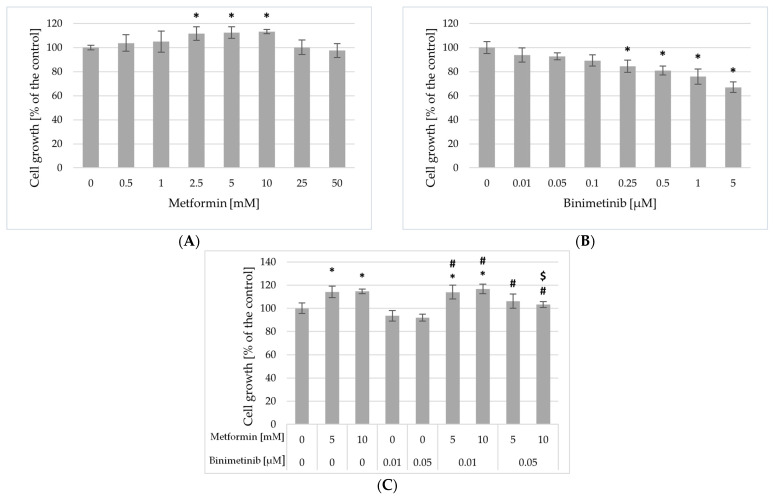
Influence of metformin (**A**), binimetinib (**B**) and both drugs (**C**) on the growth of HaCaT cells after 72 h of treatment. Data are expressed as the means ± SD and are represented as percentages of the untreated control (100% growth) of three independent experiments involving six analyses for each sample. The statistical significance of differences was assessed by one-way ANOVA followed by the post hoc Tukey’s test; * *p* < 0.05 vs. control; $ *p* < 0.05 vs. metformin alone; # *p* < 0.05 vs. binimetinib alone.

To assess the potential cytotoxic effects of both compounds on non-cancerous cells, the effect of metformin, binimetinib and their combinations on the growth of immortalized HaCaT keratinocytes was also evaluated (Figure 2). The results obtained did not show a significant impact of metformin on the inhibition of cell growth (Figure 2A). However, metformin at concentrations of 2.5; 5; and 10 mM (Met2.5; Met5; Met10) induced a slight but significant increase in cell growth. In contrast to metformin, binimetinib, depending on the concentration used, decreased on the growth of HaCaT keratinocytes (Figure 2B). Binimetinib, at the concentrations selected for the analysis of its combined effect with metformin on melanoma cells, did not induce a significant inhibitory effect on HaCaT keratinocytes growth. Binimetinib at concentrations selected to study the effects of simultaneous treatment (Bin0.01 and Bin0.05) did not cause significant changes in cell growth compared to the control. The growth inhibitory effect of binimetinib on HaCaT cells was observed in cultures treated with its concentrations ≥ 0.25 μM. Treatment of HaCaT cells with metformin and binimetinib at concentration 0.01 μM (Met5 + Bin0.01 and Met10 + Bin0.01) caused a slight but significant increase in cell growth and the effects were similar to those induced by metformin in appropriate concentrations (*p* > 0.05). However, incubation of HaCaT cells to metformin and binimetinib at concentration of 0.05 μM (Met5 + Bin0.05 and Met10 + Bin0.05) resulted in the elimination of the cell growth-stimulating effect of metformin, and there were no statistically differences compared to control cells.

### 2.3. Impact of Metformin and Binimetinib on Cells Proliferation

**Figure 3:**
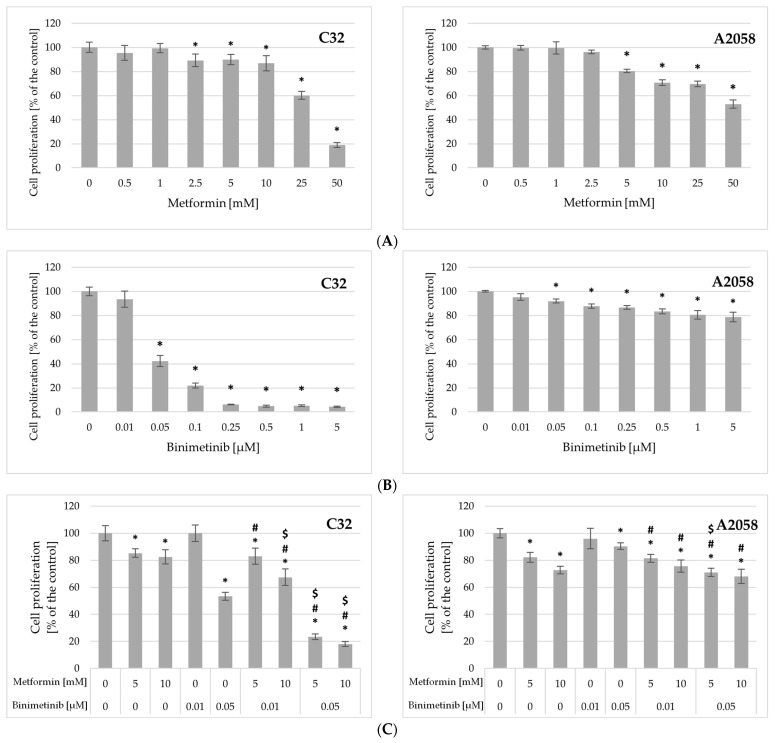
Influence of metformin (**A**), binimetinib (**B**) and both drugs simultaneously (**C**) on the proliferation of C32 and A2058 cells after 48 h of treatment. Data are expressed as the means ± SD and are represented as percentages of the untreated control (100% proliferation) of three independent experiments involving six analyses for each sample. The statistical significance of differences was assessed by one-way ANOVA followed by the post hoc Tukey’s test; * *p* < 0.05 vs. control; $ *p* < 0.05 vs. metformin alone; # *p* < 0.05 vs. binimetinib alone.

In order to evaluate the effects of the tested compounds alone and simultaneously on melanoma cell proliferation the incorporation of 3-bromodeoxyuridine (BrdU) into newly synthesized DNA after 48 h was evaluated. The obtained results have shown that metformin and binimetinib used alone decreased the incorporation of BrdU in a concentration-dependent manner (Figure 3). A significant inhibition of the proliferative activity of C32 cells was observed after incubation with metformin at concentrations ≥ 2.5 mM. In A2058 cells, a substantial decrease in DNA synthesis was achieved after treatment with concentrations ≥ 5 mM (Figure 3A). Binimetinib at the concentration 0.01 μM did not affect C32 and A2058 cell growth. A statistically significant reduction in melanoma cell growth was observed in cultures incubated with its concentrations ≥ 0.05 μM (Figure 3B) and C32 cells were more sensitive to this drug than A2058 cells. Binimetinib at concentrations of 0.25 μM and higher reduces proliferating activity of C32 cells by more than 94%, while in A2058 cell cultures the inhibition of proliferation not exceeding 21.3% (Figure 3B).

Treatment of melanoma cells with both metformin and binimetinib caused a statistically significant decrease in proliferative activity compared to the corresponding control in all combinations used (Figure 3C). In C32 cultures, the combination of the studied drugs led to greater inhibition of cell proliferation than each drug alone, except in cultures treated with Met5 + Bin0.01. The strongest inhibitory effect was observed in response to the simultaneous exposure of C32 cells to both drugs, particularly binimetinib at higher concentration (Met5 + Bin0.05 and Met10 + Bin0.05), where cell proliferation was reduced by 76.6% and 81.9% compared to the control. This effect was 30–35% stronger than that induced by binimetinib alone at a concentration of 0.05 μM. Following simultaneous exposure of A2058 cells to metformin and binimetinib, cell proliferation was significantly reduced in all treatment groups compared to both the control and binimetinib alone. However, the use of combination of both drugs did not intensify the effect compared to metformin used separately (except Met5 + Bin0.05 cultures). The findings revealed that cotreatment with metformin and binimetinib at studied concentrations affected the proliferation of melanoma cells, but it depended on the concentrations and cell line.

### 2.4. The Influence of Metformin and Binimetinib on Melanoma Cell Cycle

**Figure 4:**
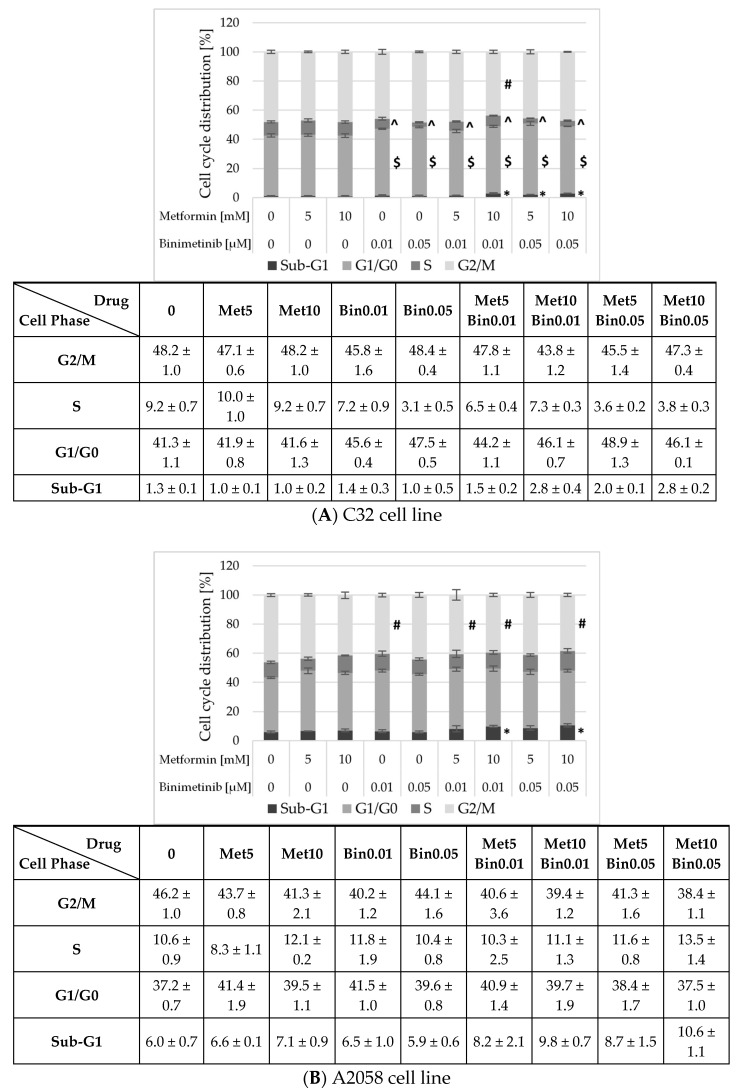
The effect of metformin and binimetinib alone and simultaneously on C32 (**A**) and A2058 (**B**) cell cycle after 24 h. For each cell line in the upper panel is shown a graph with statistical analysis, while quantitative analysis are presented in the lower panel. Data are expressed as the means ± SD and are represented as percentages of the cells in each phase of cell cycle of three independent experiments involving three analyses for each sample. The statistical significance of differences was assessed by one-way ANOVA followed by the post hoc Tukey’s test; * (subG1), $ (G1/G0), ^ (S phase), # (G2/M), *p* < 0.05 vs. control.

To further investigate the effects of metformin and binimetinib, used individually and in combination, on melanoma cells, their influence on cell cycle progression and apoptosis was evaluated by flow cytometry. Changes in the cell cycle distribution are presented in Figure 4. Metformin was shown to have no significant effect on cell cycle distribution in studied melanoma cells. Binimetinib at both concentrations (0.01 and 0.05 μM) caused a significant increase in the population of C32 cells in the G1/G0 phase and a decrease in the population of C32 cells in the S phase, compared to control (Figure 4A). Incubation of C32 cells with both drugs (Met10 + Bin0.01, Met5 + Bin0.05, and Met10 + Bin0.05) increased the population of cells in the G1/G0 phase with a corresponding reduction in the S phase. In addition, the number of cells in the sub-G1 phase was increased. The effects of studied compounds on A2058 cell cycle distribution were weaker (Figure 4B). The percentage of G2/M cell cycle phase was significantly down-regulated while that of cells in the Sub-G1 phase was increased after incubation with metformin at the concentration of 10 mM and binimetinib (Met10 + Bin0.01 and Met10 + Bin0.05).

### 2.5. The Influence of Metformin and Binimetinib on the mRNA Expression of Genes Encoding Proteins Involved in Cell Proliferation and Apoptosis Processes

Quantitative RT-PCR analysis was conducted to assess the impact of metformin and binimetinib on the transcription levels of *CCND1* and *CDNK1B* genes encoding key proteins of cell cycle, i.e., cyclin D1 and p27, respectively. The expression of *BAX*, *BCL-2* genes in relation to apoptotic pathways has also been analyzed.

**Figure 5:**
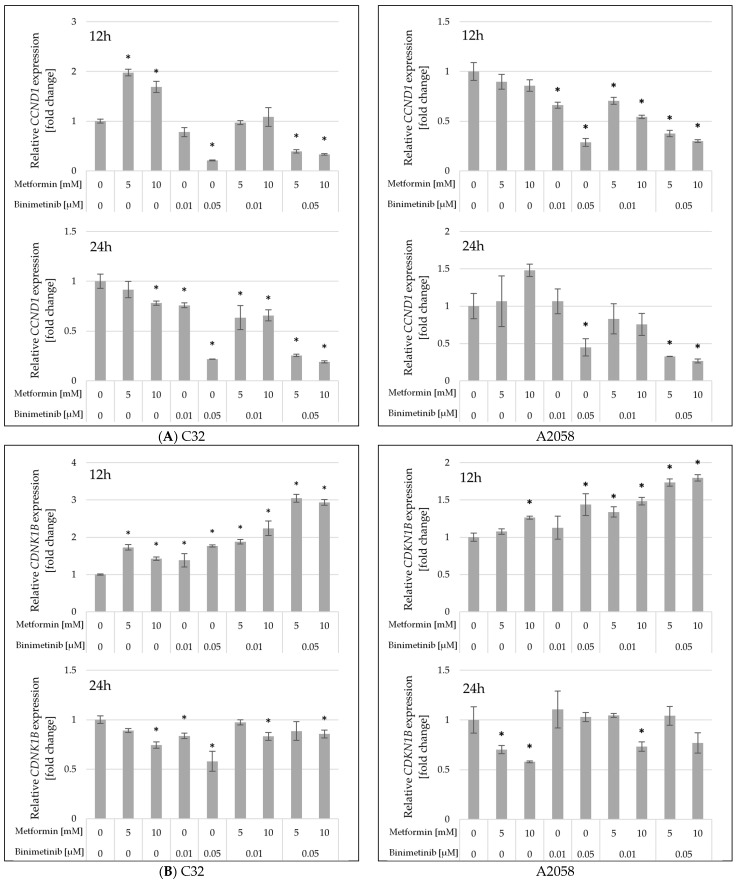
Expression of *CCND1* (**A**) and *CDKN1B* (**B**) mRNAs in C32 and A2058 melanoma cells after 12 and 24 h of metformin and binimetinib treatment. Data are expressed as the means ± SD and presented as the fold change in mRNA levels relative to the untreated control, based on three independent experiments, each analyzed in triplicate. The statistical significance of differences was assessed by one-way ANOVA followed by the post hoc Tukey’s test; * *p* < 0.05 vs. control.

Cyclin D1 is pivotal for the cell’s transition from the G1 phase to the S phase of the cell cycle. The effects of metformin and binimetinib individually and in combinations on *CCND1* gene expression in C32 and A2058 cells are shown in Figure 5A. At 12 h, a marked reduction in *CCND1* gene expression was observed in C32 cells only in cultures treated with binimetinib at a concentration of 0.05 μM both used alone and with metformin (Met5 + Bin0.05 and Met10 + Bin0.05). A longer incubation time of up to 24 h resulted in a statistically significant reduction in *CCND1* mRNA levels in all C32 cultures studied except those exposed to metformin at a concentration of 5 mM. The most pronounced decrease in *CCND1* gene expression was observed in C32 cultures treated with binimetinib at a concentration of 0.05 mM, as well as those exposed to Met5 + Bin0.05 and Met10 + Bin0.05. In A2058 melanoma cells metformin had no effect on the transcription of *CCND1* gene at 12 and 24 h. However, exposure of A2058 cells to binimetinib alone and together with metformin resulted in a statistically significant reduction in *CCND1* levels compared to control, and this effect was more pronounced after 12 h of incubation.

*CDKN1B* is a gene that codes for the p27 protein, a key cell cycle gatekeeper regulator that causes cell cycle arrest in G1 phase. In both melanoma cell lines, the most pronounced changes in *CDKN1B* mRNA levels were observed in treated cells at 12 h (Figure 5B). Prolonged incubation (up to 24 h) led to a reduction in *CDKN1B* gene expression to levels similar to the control. Exposure of C32 cells to metformin and binimetinib individually and in combination resulted in an increase in *CDKN1B* mRNA compared to untreated cells at 12h. Simultaneous exposure of C32 cells to metformin and binimetinib at a concentration of 0.05 μM (Met5 + Bin0.05 and Met10 + Bin0.05) resulted in a 3.1-fold and 2.9-fold increase in *CDKN1B* gene expression compared to the control. In A2058 cells, metformin and binimetinib at lower concentrations had no influence on *CDKN1B* expression at 12 h. However, higher concentrations of these drugs (Met10 and Bin0.05) caused a marked increase in *CDKN1B* mRNA levels compared to the control. The significant increase in *CDKN1B* expression was also observed at 12 h in all A2058 cultures incubated with both drugs simultaneously.

The observed changes in the expression of the *CCND1* and *CDKN1B* genes may indicate their possible impact on cell cycle arrest and decreased melanoma cells proliferation potential.

**Figure 6:**
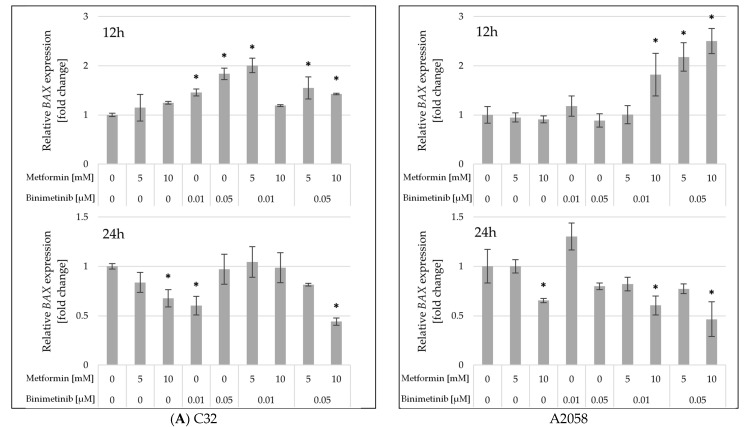

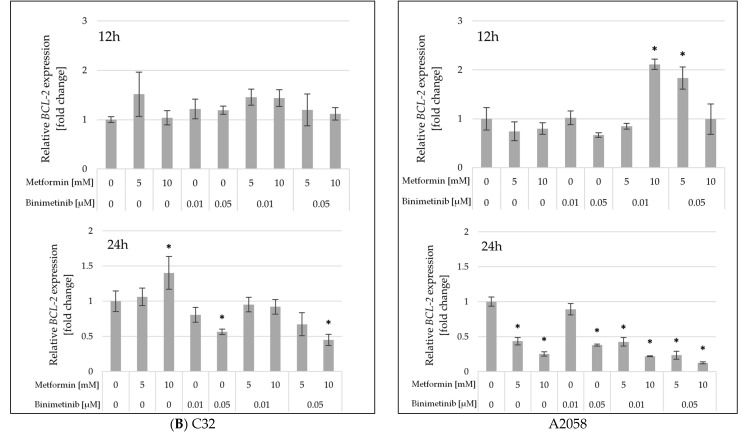
Expression of *BAX* (**A**) and *BCL-2* (**B**) mRNAs in C32 and A2058 melanoma cells after 12 and 24 h of metformin and binimetinib treatment. Data are expressed as the means ± SD and presented as the fold change in mRNA levels relative to the untreated control, based on three independent experiments, each analyzed in triplicate. The statistical significance of differences was assessed by one-way ANOVA followed by the post hoc Tukey’s test; * *p* < 0.05 vs. control.

Next, the effect of studied drugs on the expression of genes encoding proteins involved in the process of apoptosis was evaluated. The level of mRNA of the *BAX* gene in melanoma cells treated with metformin and binimetinib, alone or in combination, is shown in Figure 6A. A statistically significant increase in *BAX* gene transcript levels was observed only at 12 h. In the C32 cell line, metformin did not induce a marked increase in *BAX* gene expression, unlike binimetinib. Significant upregulation relative to control was detected in cultures treated concurrently with both drugs, except for those treated with the Met10 + Bin0.01. In the A2058 cell line, a significant increase in *BAX* gene expression was observed compared to the control in cells treated with Met10 + Bin0.01, Met5 + Bin0.05, and Met10 + Bin0.05 after 12 h.

The expression of the anti-apoptotic gene *BCL-2* was also evaluated (Figure 6B). In the C32 cell line, no statistically significant changes in its expression were observed, except in cells treated for 24 h with metformin (Met10), binimetinib (Bin0.05), and the combination Met10 + Bin0.05. In the A2058 cell line, an increase in the expression of the BCL-2 gene was observed after 12 h in cells treated with Met10 + Bin0.01 and Met5 + Bin0.05. However, extending exposure to the compounds tested to 24 h resulted in a statistically significant decrease in *BCL-2* gene expression in all cultures, except those treated with binimetinib in a concentration of 0.01 μM.

### 2.6. Impact of Metformin and Binimetinib on DNA Fragmentation

**Figure 7:**
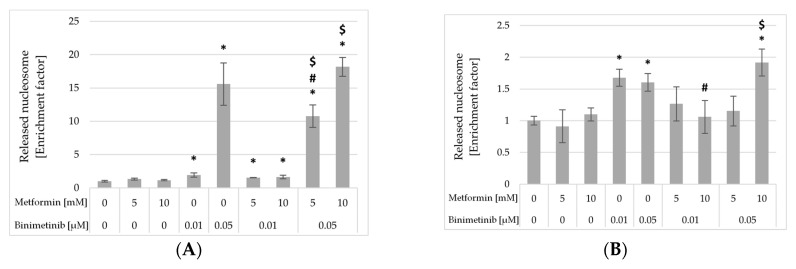
Effect of metformin and binimetinib on the induction of apoptosis in C32 (**A**) and A2058 (**B**) cells after 24 h treatment was presented as an enrichment factor, i.e., fold increase in cytoplasmatic mono and oligonucleosomes vs. control (value for control = 1). Data represent the mean ± SD of three independent experiments involving six analyses for each sample. The enrichment factor for the control is 1. The statistical significance of differences was assessed by one-way ANOVA followed by the post hoc Tukey’s test; * *p* < 0.05 vs. control; $ *p* < 0.05 vs. metformin alone; # *p* < 0.05 vs. binimetinib alone).

To estimate the impact of metformin and binimetinib on melanoma cell apoptosis, Cell Death Detection ELISA^PLUS^ assay was performed. This method enables the evaluation of DNA degradation measured by the increase in mono- and oligonucleosomes in the cytoplasm of cells during apoptosis. Cultures of C32 and A2058 cells were incubated with both drugs separately and simultaneously for 24 h (Figure 7). Subsequently, the amounts of mono- and oligonucleosomes present in the cytoplasm were measured. The analysis conducted did not reveal any significant effect of metformin on the enhancement of DNA fragmentation in the studied melanoma cell lines. In C32 melanoma cells (Figure 7A), a statistically significant increase in the amount of cytoplasmatic mono- and oligonucleosomes was observed after exposure to binimetinib alone, as well as metformin combined with binimetinib. The greatest, 15.6- and 18.2-fold increase in the enrichment factor, was observed in culture exposed to Bin0.05 as well as Met10 + Bin0.05, respectively. As shown in Figure 7B, a statistically significant increase in the amount of mono- and oligonucleosomes levels was observed in A2058 cultures upon treatment with binimetinib alone and Met10 + Bin0.05.

## 3. Discussion

Malignant melanoma is characterized by a high metastatic potential and high resistance to pharmacological treatment, which constitutes the sole available treatment modality in advanced stages of the disease [7]. Significant progress in the treatment of melanoma has been made following the introduction of immune-checkpoint inhibitors and targeted therapies (especially BRAF and MEK inhibitors) [30]. Kinase inhibitors are often used in combination therapy that involves at least two agents targeting different proteins within intracellular signal transduction pathways, improving treatment efficacy and reducing the development of cancer cell resistance resulting from excessive activation of alternative signaling pathways [31]. Binimetinib is a drug originally approved for the treatment of malignant melanoma harboring the *BRAF V600* mutation in combination with encorafenib [32], as well as non-small cell lung cancer [33]. The *BRAF V600* mutation leads to excessive activation of Ras/Raf kinases within the MAPK pathway, which in turn directly activates MEK, the molecular target of binimetinib. The presence of primary or acquired resistance in melanomas limits the therapeutic efficacy of binimetinib as a monotherapy, as MEK inhibition can lead to the activation of compensatory intracellular signaling pathways, such as the PI3K/AKT/mTOR cascade [34].

Researchers are constantly looking for combinations of anticancer drugs with new compounds. Consequently, an emerging approach to the development of novel cancer therapies is the repurposing of pharmacological agents originally approved for the treatment of non-oncological conditions. One of the drugs with potential anticancer properties is metformin, a hypoglycemic agent. It is believed to have anticancer activity derived from multifaceted biological activity, and suppression of the phosphatidylinositol 3-kinase (PI3K)/AKT cascade likely represents a key mechanism [21]. Dysregulation of the PI3K/AKT/mTOR pathway is a hallmark of numerous malignancies, driving increased cellular proliferation and inhibition of apoptotic processes, while also modulating other intracellular signaling networks. The PI3K/AKT/mTOR pathway is also one of the main transduction pathways that undergo uncontrolled activation in melanoma cells [35]. Considering that the PI3K/AKT/mTOR pathway may serve as an alternative signaling cascade to the MAPK pathway, contributing to melanoma resistance to MAPK pathway inhibitors such as binimetinib, metformin may potentially mitigate primary cellular resistance to these inhibitors and also suppress the emergence of acquired resistance [34].

Therefore, this study aimed to estimate the impact of metformin and binimetinib, applied as a single agent and in combination, on the growth, proliferation, and apoptosis of melanoma cell lines.

Preliminary in silico studies were evaluated with the ChemDIS-Mixture software to estimate the probability of pharmacological interactions of metformin and binimetinib administered simultaneously. The studies confirmed that such a combination has a high probability of efficacy in various cancers, including melanoma. Moreover, analysis suggested that the observed effect of co-treatment of cells with both drugs could be associated with their interaction with 36 signaling pathways. Many of these are associated with intracellular pathways, including those that are frequently overactivated in cancer cells and involved in tumorigenesis, such as the MAPK and VEGF pathways, as well as the ErbB pathway. Furthermore, computational analysis suggests that both agents can influence the expression of 61 genes, also critical for cellular function and development, including those involved in DNA replication, transcription, and protein phosphorylation. Although ChemDIS-Mixture serves as a valuable tool for predicting potential interactions when co-administering two or more drugs, it is essential to validate these results through experiments. The combined effect of metformin and binimetinib has been investigated so far only in two studies [36,37]. The articles cited provided evidence that the combination of both drugs may be synergistic. Moreover, Ryabaya et al. [36] suggested that the mechanism of metformin and binimetinib synergy in melanoma cells was associated with activation of *p*-AMPKα and decreased *p*-ERK, but not with altered *p*-mTOR. It is consistent with our in silico analysis, which showed that the MAPK pathway seems to be important in the interaction of metformin and binimetinib.

Some in vitro studies have evaluated that metformin, when administered concurrently with kinase inhibitors used in cancer treatment, may show a synergistic inhibitory effect against cancer cells. The synergistic anticancer effect of metformin in combination with trametinib (MEK inhibitor) has been demonstrated in ovarian cancer cells [38]. Other studies have shown that metformin synergistically enhances the cytotoxic effect of imatinib (tyrosine kinase inhibitor) [39] as well as BEZ235 (an experimental dual inhibitor of PI3K and mTOR kinases) [40] in colon cancer cells.

In the current study, the effect of metformin and binimetinib alone and simultaneously on the growth of two melanoma cell lines that exhibit differential sensitivity to protein kinase inhibitors was studied. Metformin exerted significant growth-inhibitory effects on both melanoma cell lines with IC50 values of 36.1 mM (C32) and 18 mM (A2058). Several studies have established the effect of metformin on melanoma cell growth. Tomic et al. [41] showed a dose-dependent decrease in the number of melanoma cells A375, WM9, SKMel28, and G361 after metformin treatment in the concentration range of 1–10 mM. In addition, 5- and 10-mM metformin significantly decreased the number of cells of melanoma cells isolated from the patient. In contrast, human melanocytes were resistant to metformin treatment. Tseng et al. [42] demonstrated that metformin, at concentrations ranging from 1 to 10 mM, significantly reduced the number of A2058 and A375 melanoma cells in a dose- and time-dependent manner, after exposure periods of 24 to 72 h. The cytotoxic properties of metformin at concentrations of 5, 10 and 20 mM were also demonstrated in C32 melanoma cell line [43].

As observed in this study, binimetinib also inhibited the growth of melanoma cells in both cell lines tested. The most pronounced effect was exerted against C32 cells (IC50 = 0.16 μM), while for A2058 cells the IC50 value was 1.52 μM. The observed differences in the activity of kinase inhibitors targeting the Ras/Raf/MEK/ERK signaling pathway against melanoma cells are consistent with findings reported in other studies [44]. Thumar et al. [45] studied the effect of binimetinib on growth patient-derived metastatic melanoma whose tumors harbored *BRAF* and *NRAS* mutations. They showed that binimetinib, in the 0.01–1 μM concentration range, inhibited the growth of melanoma cells in a dose-dependent manner; however, the effect depended on the type of cells.

In the presented study, the effect of metformin on the growth of the HaCaT cell line, used to assess the skin toxicity caused by various agents, was also studied. Metformin at all concentrations used did not inhibit the growth of HaCaT keratinocytes. It suggests that melanoma cells may exhibit greater susceptibility to metformin compared to non-cancer cells. Exposure of keratinocytes to binimetinib resulted in growth inhibition, although this effect was observed only at higher concentrations (≥0.25 μM) compared to melanoma cells, indicating a lower sensitivity of non-cancerous cells to the suppressive effects of binimetinib.

Liu et al. [46] showed that metformin exerted time- and dose-dependent effects on the growth of HaCaT cells; at concentrations of up to 10 mM it did not influence their growth after 72 h. Other studies showed that metformin at concentration ≥ 25 mM significantly decreases HaCaT cell growth [47]. However, neither of the studies cited demonstrated that metformin, at concentrations ≤ 10 mM over a 72 h period, exhibited inhibitory activity against HaCaT keratinocytes, which is consistent with our findings. To our knowledge, no studies have been published on the influence of binimetinib on HaCaT cells. Nevertheless, studies by Niessner et al. [48] showed that binimetinib and encorafenib alone and in combination induce only weak growth inhibition and no significant levels of apoptosis in normal skin cells (fibroblasts, keratinocytes, and melanocytes). Other studies reveal that binimetinib did not negatively affect human epidermal melanocytes HEMn-MP [49].

The presented study also evaluated the effects of metformin and binimetinib used simultaneously on melanoma cells. Analysis of the combined impact of both drugs on cell growth revealed that the combination of metformin and 0.05 μM binimetinib (Met5 + Bin0.05 and Met10 + Bin0.05) had a synergistic effect on the C32 cell growth and CI values were 0.52 and 0.56, respectively. Although A2058 cells were less sensitive to these combinations, synergism (Met5 + Bin0.05, CI = 0.68) or an additive effect (Met10 + Bin0.05, CI = 0.95) was observed. In particular, combined treatment of HaCaT keratinocytes with metformin and binimetinib did not result in growth inhibition. Ryabaya et al. [36] demonstrated a synergistic effect of metformin and binimetinib, showing that exposure of six melanoma cell lines, including those lacking *BRAF* mutations, to metformin (1 mM) and binimetinib (2 μM) resulted in at least 15% greater inhibition of cell proliferation compared to the effects of each drug used individually. This effect is further supported by the combination index (CI) values reported in the study, which ranged from 0.15 to 0.97 for the applied combination of both drugs. Lee et al. [37] provided evidence that the combination of metformin and binimetinib has synergistic effect on melanoma cells. The combined treatment of G361 melanoma cells with metformin (0.5–2 mM) and binimetinib (0.02–0.08 μM) resulted in a more pronounced reduction in cell viability compared to the effect of each agent administered individually, and the combination index (CI) values ranged from >0.8 to <1.2, depending on the concentrations used.

The findings of our study indicated that the concomitant administration of metformin and binimetinib may have a synergistic repressive effect; therefore, more detailed studies were performed to understand the molecular mechanisms underlying the melanoma growth inhibition effect.

Subsequent evaluation of the influence of metformin and binimetinib on the proliferative activity of C32 and A2058 melanoma cells showed that both drugs possess the ability to inhibit cell proliferation, which may represent one of the mechanisms underlying the observed reduction in cell growth. In particular, binimetinib alone exerted a markedly stronger inhibitory effect on the proliferation of C32 cells compared to A2058 cells. The difference in the anti-proliferative activity of the studied drugs against melanoma cells became evident when they were applied simultaneously. The proliferation of C32 cells, which showed a greater sensitivity to binimetinib, was significantly more inhibited after co-treatment with metformin and binimetinib than when the cells were exposed to either drug alone. In addition, a cell cycle analysis of the cells was performed using flow cytometry. Metformin alone did not significantly affect changes in the distribution of the melanoma cell cycle. In contrast, binimetinib induced changes characteristic of inhibited proliferation processes in the C32 cell line, resulting in an increased proportion of cells in the G0/G1 phase and a decreased proportion in the S phase. In A2058 cells, at a lower concentration, binimetinib caused an increase in the percentage of cells in the G2/M phase. These effects, an increase the cell population in phase G0/G1 with a decrease in the cell population in S phase, were also observed when C32 cells were exposed to metformin and binimetinib together. In particular, only with combined exposure of cells to the investigated compounds was an increase in the percentage of apoptotic cells (sub-G1) observed, both in the C32 and A2058 cell lines. Tseng et al. [42] observed that metformin at a concentration of 5 mM increases the number of cells in the S and G2/M phases with a concomitant decrease in the G0/G1 phase, which was not observed in our study. They suggested that metformin significantly suppressed the melanoma cell growth by inducing cell cycle arrest in the G2/M phase; however, they analysed the cell cycle after 72 h, while in the presented study the analysis was performed after 24 h. Additionally, it should be noted that they observed a high sensitivity of A375 cells, while the effects observed in the A2058 cell line were much weaker. Other studies indicate that metformin induces an increase in the proportion of A375 and other 5 patient-derived melanoma cells in the G1/G0 phase and a decrease in the S and G2/M phases after 24 h of treatment [36]. An increase in the sub-G1 melanoma cell population after exposure to binimetinib was observed in other studies on melanoma cells treated with a higher binimetinib concentration (0.5 μM) [49], as well as with 0.1 and 1 μM for a longer exposure time (72 h) [50]. Similarly, to what we observed in C32 cell lines, binimetinib treatment in other melanoma cell lines has been shown to lead to an increased proportion of cells in the G1/G0 population and a decrease in S phase population as well as a decrease in the G2/M cell population [51]. Another study revealed that combined exposure of melanoma cells to metformin (1 mM) and binimetinib (2 μM) showed a reduced number of cells undergoing S phase replication compared to the control and led to G0/G1 arrest through the cyclin D/CDK4/CDK6 pathway [36].

To understand the possible molecular mechanisms underlying the inhibition of melanoma cell growth induced by metformin and binimetinib, the activity of genes encoding key proteins involved in the regulation of the cell cycle (*CCND1* and *CDKN1B*) and apoptosis (*BAX* and *BCL-2*) was evaluated. The expression of the *CCND1* gene encoding cyclin D1 after 24 h decreased in C32 cells treated with metformin (10 mM), binimetinib, and with the combination of both drugs. In A2058 cells such an effect was observed only in cells exposed to binimetinib (0.05 μM) alone and combined with metformin. Simultaneously, it was observed increased expression of *CDKN1B* gene encoding p27 protein, a key cell cycle gatekeeper regulator. This effect was observed in both C32 cells treated with the drugs studied alone and in combination, as well as in A2058 cells treated with metformin (5 mM), binimetinib (0.05 μM) alone and together for 12 h. The results obtained suggest that growth inhibition may be related to the disruption of melanoma cell cycle and support the antiproliferative effects of binimetinib on melanoma cells, along with a possible enhancement of this effect as a result of simultaneous exposure of cells to metformin and binimetinib. However, the proposed mechanisms should be confirmed in further studies.

Other experimental studies have demonstrated that metformin increases *CCND1* expression in A2058 melanoma cells, which is consistent with our findings in both cell lines [42]. However, some published studies showed that both metformin (1 mM) and binimetinib (2 μM) caused a decrease in cyclin D1 expression in melanoma cells after 24 h [36]. The effect of metformin and binimetinib on the expression of key cell cycle regulators was only studied by Ryabaya et al. [36]. They showed that treatment of cells with both drugs reduce the expression levels of cyclin D1, CDK4 and CDK6 in melanoma cells, and their results are consistent with those of our study.

Analysis of the expression of the proapoptotic *BAX* gene did not show any upregulation in C32 and A2058 cells in response to metformin treatment. Binimetinib alone enhanced *BAX* expression after 12 h in C32 cells. However, combined exposure of cells to both drugs led to significant changes in *BAX* mRNA levels in both studied melanoma cells, which may suggest stimulation of the apoptotic pathway. In addition, a decrease in the expression of the proapoptotic *BCL-2* gene was observed in A2058 after 24 h of treatment with metformin and binimetinib, as well as in C32 cells treated with binimetinib (0.05 μM) and the combination Met10 + Bin0.05. Previously published studies also described the upregulation of *BAX* and the downregulation of *BCL-2* expression levels in G361 and SK-MEL-2 melanoma cells treated with 0.5 μM binimetinib [49].

To confirm the pro-apoptotic influence of binimetinib alone and with metformin on melanoma cells, the formation of nucleosomes, markers of apoptotic death, was evaluated. Analysis revealed that metformin alone did not induce DNA fragmentation processes in both studied melanoma cell lines. Binimetinib, on the other hand, significantly induced an increase in the level of oligonucleosomal DNA fragmentation and that process was much stronger in C32 cells than in A2058 cells. Significantly increase in the amount of mono- and oligonucleosomes were also observed in C32 cultures exposed to metformin and binimetinib as well as in A2058 cultures exposed to Met10 + Bin0.05. Based on the enrichment of nucleosomes in the cytoplasm, it can be suggested that simultaneous treatment of cells with metformin and binimetinib induce apoptosis in melanoma cells. The results of DNA fragmentation obtained by the ELISA method indicate induction of apoptosis, which was not detected in cell cycle analysis. It seems that these apparent differences may be explained by the fact that during the early stage of apoptosis, if the cell membrane retains integrity, the total amount of DNA in the cell may decrease only slightly. Therefore, at that stage of apoptosis, dying cells can be difficult to detect based on cell DNA content measurement (detection of subG1 cells). The usage a more specific and sensitive ELISA assay for oligonucleosome detection enables determination of DNA degradation, even if total DNA content in cells has not yet begun to decrease significantly. The pro-apoptotic action of binimetinib observed in the presented study is consistent with other reports. Binimetinib (0.5 μM) increased the level of cleaved caspase-3 in G361 and SK-MEL-2 melanoma cells when used alone and in combination with curcumin [49]. Furthermore, binimetinib also promoted necrosis in those cells. Ryabaya et al. [51] also confirmed induction of apoptosis by the TUNEL method in melanoma cells treated with binimetinib in a concentration of 2 μM. The capability of binimetinib alone and in combination with metformin to activate melanoma cell apoptosis was also confirmed in studies performed on patient-derived melanoma cells [36].

The presented study reveals that the combination of metformin and binimetinib may have a promising anticancer effect on melanoma cells. It has been demonstrated that metformin can enhance the anticancer activity of binimetinib against melanoma cells that differ in their sensitivity to anticancer agents. The results presented, in line with reports from other published studies, suggest that metformin may itself exhibit cytotoxic activity against melanoma cells [41,42,43]. An important conclusion derived from our study is that metformin, in combination with binimetinib, may demonstrate antiproliferative and pro-apoptotic activity.

Due to the inherent resistance of melanoma to pharmacological treatments, as well as the rapid development of resistance shortly after the initiation of therapy with targeted molecular agents, metformin could prove to be an effective drug in enhancing the efficacy of pharmacological melanoma treatment [7,34]. Recently it has been shown that metformin enhanced the anticancer activity of MEK kinase inhibitors such as binimetinib [36,37], trametinib [52], as well as EGFR kinase inhibitors like gefitinib in lung cancer cells [53]. Improved efficacy treatment in lung cancer using EGFR kinase inhibitors after metformin administration has been confirmed in clinical studies [29,54]. It should be noted that not all clinical trials that evaluated the efficacy of combined therapy with metformin and anticancer drugs in the protein kinase inhibitor class have demonstrated significant therapeutic benefits [55]. More research is needed to better determine the potential anticancer effects of metformin against melanoma. These studies should also take into account the molecular characteristics of different types of cancer, which influence the anticancer efficacy of metformin and MAPK pathway kinase inhibitors, as well as the combined anticancer activity of these compounds when used together.

In summary, the findings suggest that the combination of metformin and binimetinib may have a promising anticancer effect on melanoma cells. However, further experiments are essential to confirm the anticancer effect of cotreatment with metformin and binimetinb against melanoma cells and to evaluate the underlying mechanism of action of the combination.

## 4. Materials and Methods

### 4.1. In Silico Drugs Interaction Analysis

In silico studies were performed to determine the possible effects of the simultaneous use of metformin and binimetinib in the treatment of melanoma cancer. The analysis was performed in the online Chem-DIS Mixture version 2.4 software (https://cwtung.nhri.edu.tw/chemdis/ (accessed on 30 June 2025)), which allows to determine the potential effects of the combined use of tested compounds based on their affinity to receptors and nonreceptor proteins [56]. The analysis performed is based on hypergeometric tests with the Benjamini–Hochberg method for corrections with a corrected *p* value < 0.05. The analysis allows for the identification of proteins and pathways affected by the compounds studied. The software also identifies diseases in which the combined use of two or more investigational drugs may provide the greatest therapeutic benefit. For the prioritization of potential interaction effects, the joint *p*-value (P _Joint_) was used, where *p* is the value for drug alone.

### 4.2. Cell Lines and Culture Conditions

The study was carried out on the amelanotic melanoma C32 cell line and the melanotic melanoma A2058 cell line. Both melanoma cell lines harbor the *BRAFV600* mutation, which is commonly associated with aberrant activation of the MAPK pathway in melanoma. The study was also performed on keratinocytes HaCaT cell line that served as a model of normal skin cells. All cell lines were obtained from LGC Standards (Łomianki, Poland) originating from the American Type Culture Collection (ATCC^®^, Rockvile, MD, USA). Cancer cells were cultured in MEM medium (Sigma-Aldrich, St. Louis, MO, USA), while keratinocytes were cultured in DMEM medium (Sigma-Aldrich) both supplemented with 10% fetal bovine serum (BioWest, Nualillé, France), 100 U/mL penicillin and 100 µg/mL streptomycin (Sigma-Aldrich) at 37 °C in humidified 5% CO_2_ incubator.

### 4.3. Preparation of Drug Solutions

Metformin (Met) (Sigma-Aldrich) was dissolved in molecular grade water to obtain the stock solution 1 M. Binimetinib (Bin) (Cayman Chemical, Ann Arbor, MI, USA) was dissolved in DMSO (Sigma-Aldrich) to obtain the stock solution 66.66 mM. Before use, the stock solutions were diluted in sterile culture medium to the concentrations studied. The concentration of DMSO in the culture media was 0.01%.

### 4.4. Cell Growth Assay

To evaluate the effect of drugs studied alone and in combination on the growth of melanoma and keratinocytes cells the “In Vitro Toxicology Assay Kit, Sulforhodamine B (SRB) based” (Sigma Aldrich, St. Louis, MO, USA) was used. For this purpose, cells were seeded into 96-well plates at 1.5 × 10^3^ cells (A2058), 5 × 10^3^ cells (C32) and 2.5 × 10^3^ cells (HaCaT) per well. After 48 h, cultures were exposed to metformin (0.5–50 mM) and binimetinib (0.01–5 µM) alone and in combination. Control cultures not treated with the drugs tested were also conducted. The growth of the examined cells was measured spectrophotometrically after 72 h of exposure according to the manufacturer’s protocol and expressed as a percentage of the control. The results obtained were used to calculate the IC50 values (GraphPad Prism version 7, San Diego, CA, USA), which represents the concentration of the compounds tested that causes a 50% inhibition of cell growth. For indication of drugs interactions CompuSyn 1.0 software (ComboSyn, Inc., New York, NY, USA) was used. Following the methodology and algorithms proposed by Chou [57], the Combination Index (CI) was used. A CI value below 0.9 indicates synergism, values above 1.1 suggest antagonism, while values between 0.9 and 1.1 correspond to an additive effect.

### 4.5. Evaluation of the Cell Proliferation

To estimate the effect of metformin and binimetinib on melanoma cells proliferation ELISA “BrdU Cell Proliferation Assay” (Roche, Mannheim, Germany) tests were performed. Cells were seeded in 96-well plates at a density of 5 × 10^3^ in 200 µL medium and incubated for 48 h. Subsequently, the media were replaced with a fresh containing metformin (0.5–50 mM) and binimetinib (0.01–5 µM) alone or in combination and treated for 48 h. An amount of 20 µL BrdU solution was added to the media for the last 2 h of incubation. After the media was removed, cells were subjected to fixation and DNA denaturation using FixDenat solution. The formation of immune complexes was achieved by applying antibodies conjugated with peroxidase. The incorporation of BrdU into cellular DNA was quantified by measuring absorbance at 450 nm, with a reference wavelength of 690 nm to correct for background (LabtechLT-5000c plate reader). The proliferation of treated cells was expressed as a percentage of untreated control cells.

### 4.6. Cell Cycle Analysis

The impact of the drugs studied on cell distribution within the cell cycle was determined using the flow cytometry technique. For this purpose, cancer cells were plated in six-well plates at a density of 7.5 × 10^4^ cells per well in 3 mL medium and incubated for 48 h to facilitate cell adhesion. Subsequently, cell cultures were exposed to Met (5 and 10 mM) and Bin (0.01 and 0.05 μM) alone and in combination for the next 24 h. Subsequently, cells were trypsinised and 1 mL of the cell suspension was transferred into test tubes. The cell DNA was stained with the Vybrant^®^ DyeCycle^TM^ Green (Thermo Fisher Scientific, Waltham, MA, USA) according to the manufacturer’s instructions. The DNA content in the cells was measured using the Guava^®^easyCyte^TM^ flow cytometer (Merck, Darmstadt, Germany). The cell cycle distribution of cells was processed by using Flowing Software 2 (Turku Bioscience, Turku, Finland).

### 4.7. Total RNA Extraction and Gene Expression Assessment

The expression of genes in colon and melanoma cancer cells was assessed using the real-time RT-qPCR technique. The expression of genes involved in cell cycle regulation (*CCND1* and *CDKN1B*) and apoptosis (*BAX*, *BCL-2*) was analyzed. Cells were seeded at a density of 7.5 × 10^5^ per 56.7 cm^2^ culture plates. Following a 48 h incubation period, cells were treated with fresh medium supplemented with the investigational compounds for 12 and 24 h. Total RNA was extracted from cells with the use of TRI Reagent (Sigma Aldrich, St. Louis, MO, USA) according to the manufacturer’s instructions. RNA extracts isolated from melanoma cell lines C32 and A2058 were purified to remove melanin prior to further analysis, as melanin could interfere with reverse transcriptase activity and disrupt subsequent assays. Purification was performed using the OneStep™ PCR Inhibitor Removal Kit (Zymo Research, Irvine, CA, USA). RNA concentration and purity were evaluated using a Shimadzu UV-1800 spectrophotometer (Shimadzu, Kyoto, Japan). The analysis of gene copy number was performed using the SensiFAST™ SYBR No-ROX One-Step Kit (Bioline, Meridian Bioscience, Cincinnati, OH, USA) on a CFX Connect Real-Time PCR Detection System (Bio-Rad, Hercules, CA, USA). The RT-qPCR thermal cycling protocol consisted of reverse transcription at 45 °C for 10 min, followed by an initial denaturation step at 95 °C for 2 min. Amplification was then performed for 45 cycles with the following conditions: 95 °C for 5 s, 60 °C for 10 s, and 72 °C for 5 s. Target genes mRNA copy numbers of the were determined using a commercially available β-actin standard (TaqMan DNA Template Reagent Kit, Invitrogen) and normalized to the total RNA content. The primers used in the analysis, the sequences of which are provided in Table 4, are commercially available (Sigma Aldrich, St. Louis, MO, USA). The results are presented as the fold change in gene expression relative to the control.

### 4.8. DNA Fragmentation Assay

To investigate the apoptosis promoting capacity of Met and Bin “Cell Death Detection ELISA^PLUS^” (Roche) was employed. This method identifies nuclear DNA fragmentation through the binding of antibodies directed against DNA and histones in mono- and oligonucleosomes, arising during apoptotic process. Melanoma cells were seeded in 96-well plates at a density of 7.5 × 10^3^ cells in 200 μL medium. After 48 h of incubation, cultures were treated with Met and Bin alone and in combination for 24 h. According to the manufacturer’s instructions, cells were lysed and subjected to centrifugation to obtain a supernatant containing nucleosomes. The presence of mono- and oligonucleosomes released into the cytoplasm was detected through the use of biotinylated anti-histone antibodies and peroxidase-conjugated anti-DNA antibodies. The absorbance was measured at 405 nm using the plate reader (LabtechLT-5000c, Labtech International Ltd., Uckfield, UK) with 490 nm serving as the reference wavelength. DNA fragmentation was assessed based on the enrichment of mono- and oligonucleosomes, associated with histones, released into the cytoplasm. According to the manufacturer’s protocol the enrichment factor was used as the parameter of apoptosis to evaluate the fold increase in DNA fragmentation in treated sample relative to the control.

### 4.9. Statistical Analysis

All experiments were performed three times. The experimental values were analyzed using the Excel system. Statistical evaluations were conducted using STATISTICA 13.3 (TIBCO Software Inc., Palo Alto, CA, USA). The data obtained were presented as mean ± standard deviation (SD). The normality of the data distribution was examined with the Shapiro–Wilk test. The one-way analysis of variance (ANOVA) test, followed by post hoc Tukey’s test was used for statistical comparisons among the groups. Results with *p*-value less than 0.05 were considered statistically significant.

## Figures and Tables

**Table 1:** Selected pathways identified as a common target of metformin (Met) and binimetinib (Bin).

Pathway Analysis				
ID	Description	Adj.P Met	Adj.P Bin	Adj.P Joint
hsa04910	Insulin signaling pathway	6.53 × 10^−16^	1.05 × 10^−3^	6.83 × 10^−19^
hsa05200	Pathways in cancer	4.48 × 10^−13^	2.15 × 10^−4^	9.65 × 10^−17^
hsa04010	MAPK signaling pathway	1.67 × 10^−7^	6.30 × 10^−4^	1.05 × 10^−10^
R-HSA-5675221	Negative regulation of MAPK pathway	8.62 × 10^−6^	2.33 × 10^−5^	2.01 × 10^−10^
hsa04012	ErbB signaling pathway	7.53 × 10^−6^	4.04 × 10^−4^	3.04 × 10^−9^
hsa04370	VEGF signaling pathway	1.86 × 10^−5^	3.18 × 10^−4^	5.92 × 10^−9^
R-HSA-2428933	SHC-related events triggered by IGF1R	1.13 × 10^−2^	9.28 × 10^−7^	1.05 × 10^−8^
R-HSA-112412	SOS-mediated signalling	3.98 × 10^−2^	6.09 × 10^−7^	2.43 × 10^−8^
hsa04722	Neurotrophin signaling pathway	3.65 × 10^−5^	9.85 × 10^−4^	3.60 × 10^−8^
R-HSA-5673001	RAF/MAP kinase cascade	4.61 × 10^−2^	1.39 × 10^−6^	6.41 × 10^−8^
R-HSA-1433557	Signaling by SCF-KIT	3.95 × 10^−3^	2.33 × 10^−5^	9.21 × 10^−8^
hsa04664	Fc epsilon RI signaling pathway	3.75 × 10^−4^	3.20 × 10^−4^	1.20 × 10^−7^
hsa04510	Focal adhesion	1.50 × 10^−5^	3.74 × 10^−2^	5.59 × 10^−7^
R-HSA-5668599	RHO GTPases Activate NADPH Oxidases	7.54 × 10^−5^	2.10 × 10^−2^	1.58 × 10^−6^
hsa04916	Melanogenesis	3.57 × 10^−3^	5.71 × 10^−4^	2.04 × 10^−6^
hsa04660	T cell receptor signaling pathway	1.37 × 10^−2^	6.38 × 10^−4^	8.76 × 10^−6^
hsa04062	Chemokine signaling pathway	3.20 × 10^−2^	3.02 × 10^−4^	9.65 × 10^−6^
hsa04650	Natural killer cell mediated cytotoxicity	1.74 × 10^−2^	1.04 × 10^−3^	1.81 × 10^−5^
hsa04912	GnRH signaling pathway	3.66 × 10^−2^	5.71 × 10^−4^	2.09 × 10^−5^
SMP00358	Fc Epsilon Receptor I Signaling in Mast Cells	3.39 × 10^−2^	6.20 × 10^−4^	2.10 × 10^−5^
R-HSA-109704	PI3K Cascade	9.88 × 10^−3^	4.79 × 10^−2^	4.73 × 10^−4^

**Table 2:** Selected genes identified as a common target of metformin (Met) and binimetinib (Bin).

Go Analysis						
ID	Description	Adj.P Met	Gene Ratio Met	Adj.P Bin	Gene Ratio Bin	Adj.P Joint
GO:0045944	positive regulation of transcription from RNA polymerase II promoter	1.25 × 10^−16^	94/615	2.71 × 10^−2^	4/18	3.39 × 10^−18^
GO:0045893	positive regulation of transcription, DNA-templated	6.82 × 10^−13^	58/615	1.20 × 10^−2^	4/18	8.17 × 10^−15^
GO:0000165	MAPK cascade	3.13 × 10^−5^	26/615	1.69 × 10^−8^	8/18	5.31 × 10^−13^
GO:0008284	positive regulation of cell proliferation	1.17 × 10^−8^	46/615	2.53 × 10^−2^	3/18	2.97 × 10^−10^
GO:0001934	positive regulation of protein phosphorylation	2.20 × 10^−8^	22/615	2.44 × 10^−2^	2/18	5.37 × 10^−10^
GO:0001077	transcriptional activator activity, RNA polymerase II core promoter proximal region sequence-specific binding	5.56 × 10^−8^	30/615	3.45 × 10^−2^	2/17	1.92 × 10^−9^
GO:0005829	cytosol	1.19 × 10^−7^	168/613	1.93 × 10^−2^	8/18	2.30 × 10^−9^
GO:0043524	negative regulation of neuron apoptotic process	1.27 × 10^−5^	18/615	2.00 × 10^−4^	4/18	2.55 × 10^−9^
GO:0000978	RNA polymerase II core promoter proximal region sequence-specific DNA binding	1.19 × 10^−6^	35/615	4.97 × 10^−2^	2/17	5.93 × 10^−8^
GO:0051721	protein phosphatase 2A binding	2.83 × 10^−6^	9/615	3.71 × 10^−2^	1/17	1.05 × 10^−7^
GO:0006468	protein phosphorylation	5.79 × 10^−6^	39/615	2.53 × 10^−2^	3/18	1.47 × 10^−7^
GO:0042149	cellular response to glucose starvation	7.77 × 10^−6^	9/615	3.70 × 10^−2^	1/18	2.88 × 10^−7^
GO:0030335	positive regulation of cell migration	2.20 × 10^−4^	19/615	8.15 × 10^−3^	3/18	1.79 × 10^−6^
GO:0032403	protein complex binding	1.50 × 10^−4^	21/615	1.87 × 10^−2^	3/17	2.81 × 10^−6^
GO:0045740	positive regulation of DNA replication	6.00 × 10^−4^	8/615	8.15 × 10^−3^	2/18	4.89 × 10^−6^

**Table 3:** Selected malignancies with the highest probability of therapeutic advantage of the combined administration of metformin (Met) and binimetinib (Bin).

DO Analysis				
ID	Description	Adj.P Met	Adj. P Bin	Adj. P Joint
DOID:3119	gastrointestinal system cancer	1.09 × 10^−20^	9.61 × 10^−3^	1.04 × 10^−22^
DOID:684	hepatocellular carcinoma	8.74 × 10^−21^	2.75 × 10^−2^	2.40 × 10^−22^
DOID:686	liver carcinoma	8.74 × 10^−21^	2.75 × 10^−2^	2.40 × 10^−22^
DOID:3571	liver cancer	2.24 × 10^−20^	2.78 × 10^−2^	6.23 × 10^−22^
DOID:10155	intestinal cancer	9.78 × 10^−17^	1.37 × 10^−3^	1.34 × 10^−19^
DOID:3856	male reproductive organ cancer	1.22 × 10^−17^	1.23 × 10^−2^	1.50 × 10^−19^
DOID:2531	hematologic cancer	2.32 × 10^−17^	8.48 × 10^−3^	1.97 × 10^−19^
DOID:0060083	immune system cancer	2.67 × 10^−17^	8.75 × 10^−3^	2.33 × 10^−19^
DOID:9256	colorectal cancer	2.05 × 10^−16^	1.37 × 10^−3^	2.80 × 10^−19^
DOID:5672	large intestine cancer	2.37 × 10^−16^	1.37 × 10^−3^	3.24 × 10^−19^
DOID:219	colon cancer	3.64 × 10^−16^	1.37 × 10^−3^	4.97 × 10^−19^
DOID:10283	prostate cancer	1.21 × 10^−17^	4.18 × 10^−2^	5.04 × 10^−19^
DOID:1324	lung cancer	8.31 × 10^−17^	7.16 × 10^−3^	5.95 × 10^−19^
DOID:1240	leukemia	1.95 × 10^−16^	4.48 × 10^−3^	8.72 × 10^−19^
DOID:0050615	respiratory system cancer	2.30 × 10^−16^	7.44 × 10^−3^	1.71 × 10^−18^
DOID:0050686	organ system cancer	5.63 × 10^−16^	1.09 × 10^−2^	6.16 × 10^−18^
DOID:3908	non-small cell lung carcinoma	1.19 × 10^−14^	2.64 × 10^−3^	3.15 × 10^−17^
DOID:3905	lung carcinoma	2.40 × 10^−14^	4.21 × 10^−3^	1.01 × 10^−16^
DOID:1749	squamous cell carcinoma	1.02 × 10^−13^	1.37 × 10^−3^	1.39 × 10^−16^
DOID:1909	melanoma	1.41 × 10^−11^	1.37 × 10^−3^	1.92 × 10^−14^

**Table 4:** Characteristics of the primers used in the experiment.

Gene	Forward Primer (5′-3′)	Reverse Primer (5′-3′)
*CCND1*	GCCTCTAAGATGAAGGAGAC	CCATTTGCAGCAGCTC
*CDKN1B*	AAAATGTTTCAGACGGTTCC	ATTCGAGCTGTTTACGTTTG
*BAX*	TCTGAGCAGATCATGAAGAC	TCCATGTTACTGTCCAGTTC
*BCL-2*	GATTGTGGCCTTCTTTGAG	GTTCCACAAAGGCATCC

## Data Availability

Data are contained within this article.

## Abbreviations

The following abbreviations are used in this manuscript:

AKT	Protein Kinase B
AMPK	5′AMP-activated protein kinase
*BAX*	Gene encoding BAX protein
*BCL-2*	Gene encoding BCL-2 protein
Bin	Binimetinib
BRAF	Protein kinase encoded by the *BRAF* oncogene, member of the MAPK signaling cascade
*BRAF*	B-raf proto-oncogene
BrdU	5-bromo-2′-deoxyuridine
*CCND1*	Gene encoding cyclin D1 protein
CDK4	Cyclin-dependent kinase 4
CDK6	Cyclin-dependent kinase 6
*CDKN1B*	Gene encoding p27 protein
CI	Combination Index
DMSO	Dimethyl sulfoxide
EGFR	Epidermal growth factor receptor
ErbB	Family of receptor tyrosine kinases, structurally related to the epidermal growth factor receptor
IC50	Inhibitory concentration
MAPK	Mitogen-activated protein kinases
MEK	Protein kinase, member of the MAPK signaling cascade
Met	Metformin
mTOR	Mammalian target of rapamycin kinase
*NRAS*	Neuroblastoma ras viral oncogene homolog
PD-1	Programmed death receptor 1
PI3K	Phosphatidylinositol 3-kinase
RT-qPCR	Quantitative reverse transcription polymerase chain reaction
SRB	Sulforhodamine B
TUNEL	Terminal deoxynucleotidyl transferase dUTP nick-end labeling
VEGF	Vascular endothelial growth factor
